# Three-Dimensional Bi-Continuous Nanoporous Gold/Nickel Foam Supported MnO_2_ for High Performance Supercapacitors

**DOI:** 10.1038/s41598-017-17872-3

**Published:** 2017-12-19

**Authors:** Jie Zhao, Xilai Zou, Peng Sun, Guofeng Cui

**Affiliations:** 1 School of Mechanical and Automotive Engineering, South China University of Technology, Guangzhou, 510640 China; 2 Key Laboratory for Polymeric Composite & Functional Materials of Ministry of Education, School of Chemistry, Sun Yat-sen University, Guangzhou, 510275 China; 3 Key Laboratory of Low-carbon Chemistry & Energy Conservation of Guangdong Province, Sun Yat-sen University, Guangzhou, 510275 China

## Abstract

A three-dimensional bi-continuous nanoporous gold (NPG)/nickel foam is developed though the electrodeposition of a gold–tin alloy on Ni foam and subsequent chemical dealloying of tin. The newly-designed 3D metal structure is used to anchor MnO_2_ nanosheets for high-performance supercapacitors. The formed ternary composite electrodes exhibit significantly-enhanced capacitance performance, rate capability, and excellent cycling stability. A specific capacitance of 442 Fg^−1^ is achieved at a scan rate of 5 mV s^−1^ and a relatively high mass loading of 865 μg cm^−2^. After 2500 cycles, only a 1% decay is found at a scan rate of 50 mV s^−1^. A high power density of 3513 W kg^−1^ and an energy density of 25.73 Wh kg^−1^ are realized for potential energy storage devices. The results demonstrate that the NPG/nickel foam hybrid structure significantly improves the dispersibility of MnO_2_ and makes it promising for practical energy storage applications.

## Introduction

The rapid development of the electronics industry has increased demands in corresponding electrical energy storage devices. Among various energy storage devices, pseudocapacitors have attracted significant interest during the past decade due to their high specific capacitance, excellent charge/recharge characteristics, and long cycling life^1^. The most widely used active electrode materials for pseudocapacitors include transition metal oxides and hydroxides such as RuO_2_^2,3^, CoO^4^, NiO^5^, and MnO_2_^6^, which possess a range of reversible oxidation states for highly efficient redox charge transfer. Among them, MnO_2_ has been regarded as one of the most promising pseudocapacitive materials for high performance supercapacitors (SCs) owing to its high theoretical specific capacitance (1370 F g^−1^), low cost, environmentally friendly nature, and natural abundance^7,8^. However, MnO_2_ electrodes often suffer from intrinsically poor conductivity (10^−5^~10^−6^ S cm^−1^)^9,10^. The respectable theoretical capacitance can only be realized in the form of thin films (ten of nanometers) or nanoparticles with a low loading amount (<10 μg cm^−2^)^11^.

To overcome the aforementioned drawback, a variety of strategies have been employed to improve the conductivity of MnO_2_. Jayan Thomas *et al*. utilized spin-on nanoprinting to print large area, well-ordered PAN nanopillar arrays for the loading of MnO_2_^12^. Various carbon based composite materials, such as carbon nanoparticles (CNPs)^13^, carbon nanotubes (CNTs)^14–18^, carbon nanowires (CNWs)^19–21^, and graphene^22–25^ have also been introduced to create hybrid materials with MnO_2_ oxides to improve the conductivity. Despite some of the improvements, researchers still suffer from complex fabricating procedures and modest capacitive behavior.

Recently, nanoporous gold (NPG) has attracted much attention for its excellent conductivity, large surface area, chemical stability, and biocompatibility. It has been regarded as a potential candidate for various areas such as supercapacitors^26,27^, sensors^28^, catalysis^29^, fuel cells^30^, and enhanced fluorescence^31^. Professor Chen *et al*. have proposed thick MnO_2_ layer on free-standing NPG films to close the theoretical gravimetric specific capacitance of MnO_2_^27^. They also fabricated these electrodes into nonaqueous symmetric supercapacitors for demonstrating the advantages of this structure^32^. However, traditional NPG films are typically obtained by etching Au-Ag alloy thin film derived from the melting method, which is unsafe and consume much energy. In addition, the highest gravimetric specific capacitance can only be gained when the MnO_2_ layer is rather thin (very low mass-loading), which is far away from commercial application^33^.

To resolve this problem, we developed a mild two-step strategy to fabricate high quality NPG films directly supported on Ni foam for the loading of MnO_2_ which acts as a high-performance supercapacitor. A room temperature electrodeposition method of the Au-Sn alloy was used to integrate the Au-Sn thin film directly onto the Ni foam, followed by selective chemical dealloying of Sn and electrodeposition of MnO_2_. The fabricating strategy is safe, facile, reproducible, and relatively inexpensive compared to other methods for preparing noble metal substrates. The hierarchical NPG/Ni foam structure is of great importance in our design of a supercapacitor as it not only ensures efficient charge/electrolytes transfer, but also provides a substrate with large surface area to disperse loaded MnO_2_ and prevent it from agglomerating. Significant improvement was observed by comparing capacitive properties of samples with and without the NPG structure. By introducing NPG structure, the electrode exhibits a capacitance of nearly 3 times higher than the one without. The proposed 3D bi-continuous metal structure may have the potential to be applied to many promising energy storage devices in which the performance is mainly limited by the low conductivity of materials.

## Results

**Figure 1:**
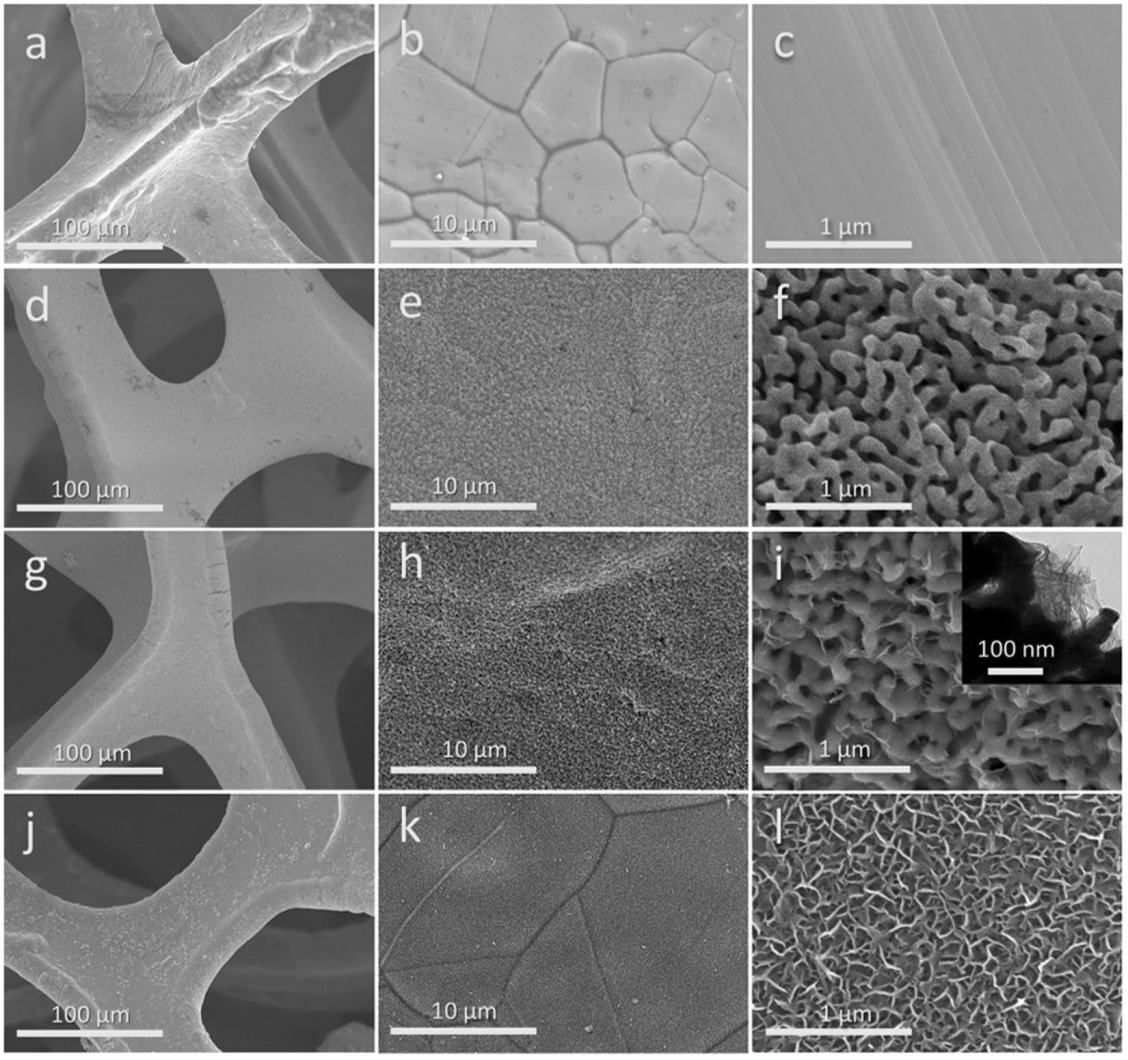
SEM images of bare Ni foam (**a**–**c**) NPG (**d**–**f**) MnO_2_/NPG (**g**–**i**) and MnO_2_/Ni foam (**j**–**l**) with different magnification. The inset in (**i**) shows the TEM image of the MnO_2_/NPG nano structure.

Figure 1 shows low and high-magnification SEM images of the MnO_2_/NPG/Ni foam (denoted as MnO_2_/NPG) and the MnO_2_/Ni foam. The Ni foam has the typical morphology of a porous framework, with a pore size of 200–300 μm (Fig. 1a–c). From Fig. 1d–f, it can be seen that a continuous thin film of NPG is uniformly coated on the Ni foam and the nanopores are of 50–150 nm. Few cracks were observed during the dealloying procedure because the initial Sn/Au ratio (about 1:1 in atomic ratio) is not enough to cause a significant shrinkage of volume. After depositing MnO_2_ on NPG, we can see that some nanosheets were loaded on NPG skeleton (Fig. 1g–i). For the sample with the MnO_2_ plating amount up to 865 μg cm^−2^ (examined by ICP), no obvious agglomeration of MnO_2_ nanosheets was observed on the surface of the NPG film. As a control group, MnO_2_ directly deposited on Ni foam presents numerous, densely-packed MnO_2_ nanosheets (Fig. j-l). In contrast, the MnO_2_/NPG (Fig. 1c and inset) illustrates a bi-continuous nanoporous structure that consists of quasi-periodic nanopores and gold ligaments, on which the MnO_2_ layer appears much more dispersive. The detailed structure of MnO_2_/NPG can also be observed in TEM image (inset in Fig. 1i).

**Figure 2:**
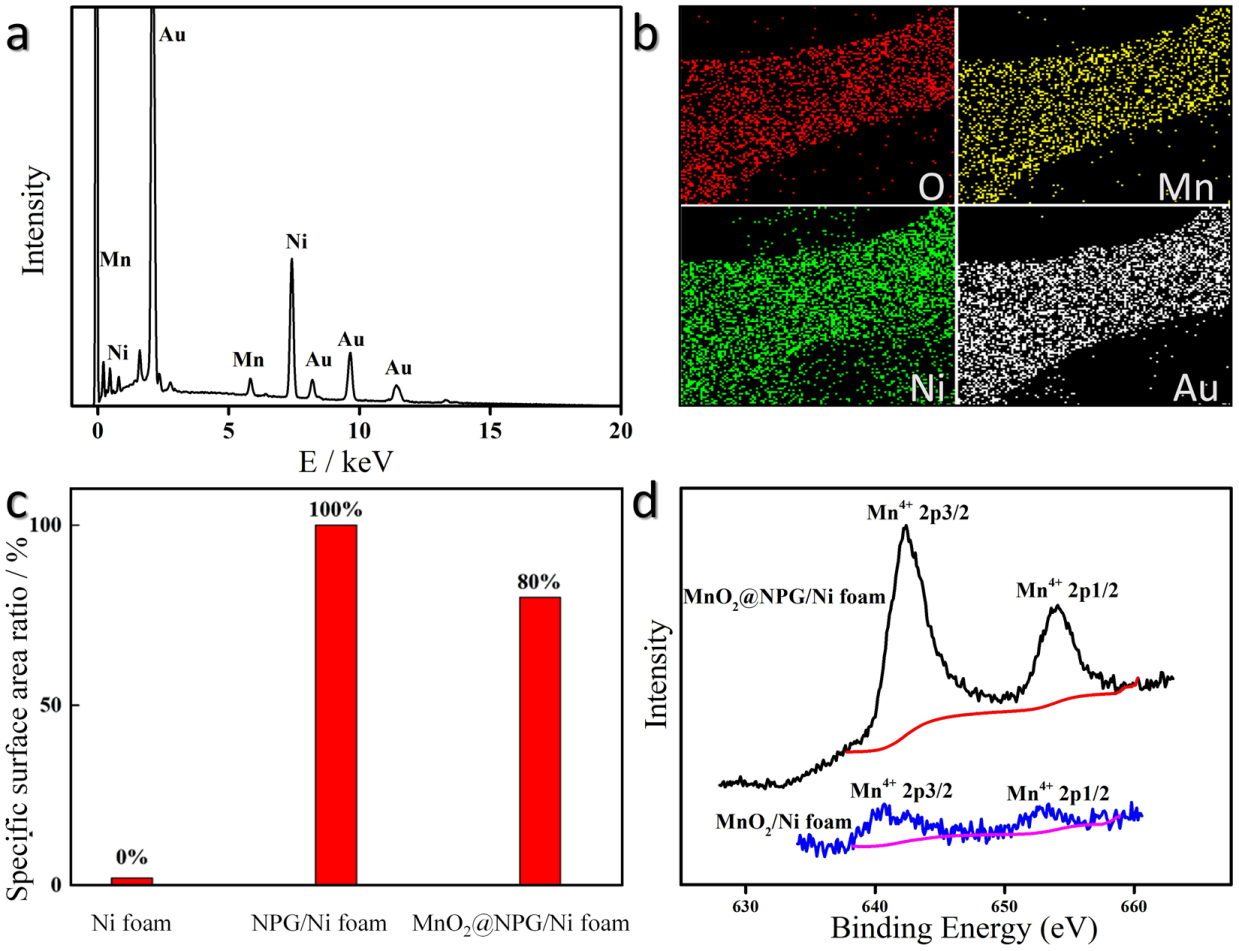
Energy-dispersive spectrum of the MnO_2_/NPG (**a**), corresponding elemental mapping images of O, Mn, Ni, and Au (**b**), comparison of the BET specific surface areas of the Ni foam, NPG/Ni foam, and MnO_2_/NPG (**c**), and XPS spectra of Mn 2p for the MnO_2_/NPG and the MnO_2_/Ni foam electrodes (**d**).

The evidence of successful hybridization can also be verified through the energy-dispersive spectrum (EDS), shown in Fig. 2a. Only three metallic elements in the MnO_2_/NPG electrode were detected. The corresponding elemental mapping images demonstrated the uniform distribution of O, Mn, Ni, and Au (Fig. 2b), which match the SEM images very well.

The BET measurement (Fig. 2c) reveals that the specific surface area of Ni foam approaches zero relative to nanoporous gold’s level (24.8 m^2^ g^−1^), and the specific surface area of MnO_2_/NPG was reduced to approximately 80% (19.84 m^2^ g^−1^) as a result of the MnO_2_ electrodeposition, which is in good agreement with SEM and TEM results.

The XPS spectra of the MnO_2_/NPG and MnO_2_/Ni foam electrodes are shown in Fig. 2d. It mainly consists of two distinct peaks centred at 642.8 eV and 654.6 eV, which are respectively ascribed to Mn 2p3/2 and Mn 2p1/2^34^. In this work, the signals of Mn 2p are most likely caused by the Mn^4+^ chemical state as MnO_2_. In addition, the Mn^4+^ peaks of the MnO_2_/NPG distinctly shift to higher energies than those of the MnO_2_/Ni foam, indicating the strong chemical interaction between Au and MnO_2_^6,35^. For a deposition time of 20 min, the amounts of loaded MnO_2_ are almost the same for the two electrodes, but the peaks’ intensity of Mn^4+^ in the MnO_2_/NPG is much higher, revealing that the presence of nanoporous gold significantly improved the dispersibility of MnO_2_ on the substrate^36^. These results also suggest that the introduction of nanoporous gold onto nickel foam may contribute to enhanced ion and electron diffusion, resulting in a high-rate performance.

**Figure 3:**
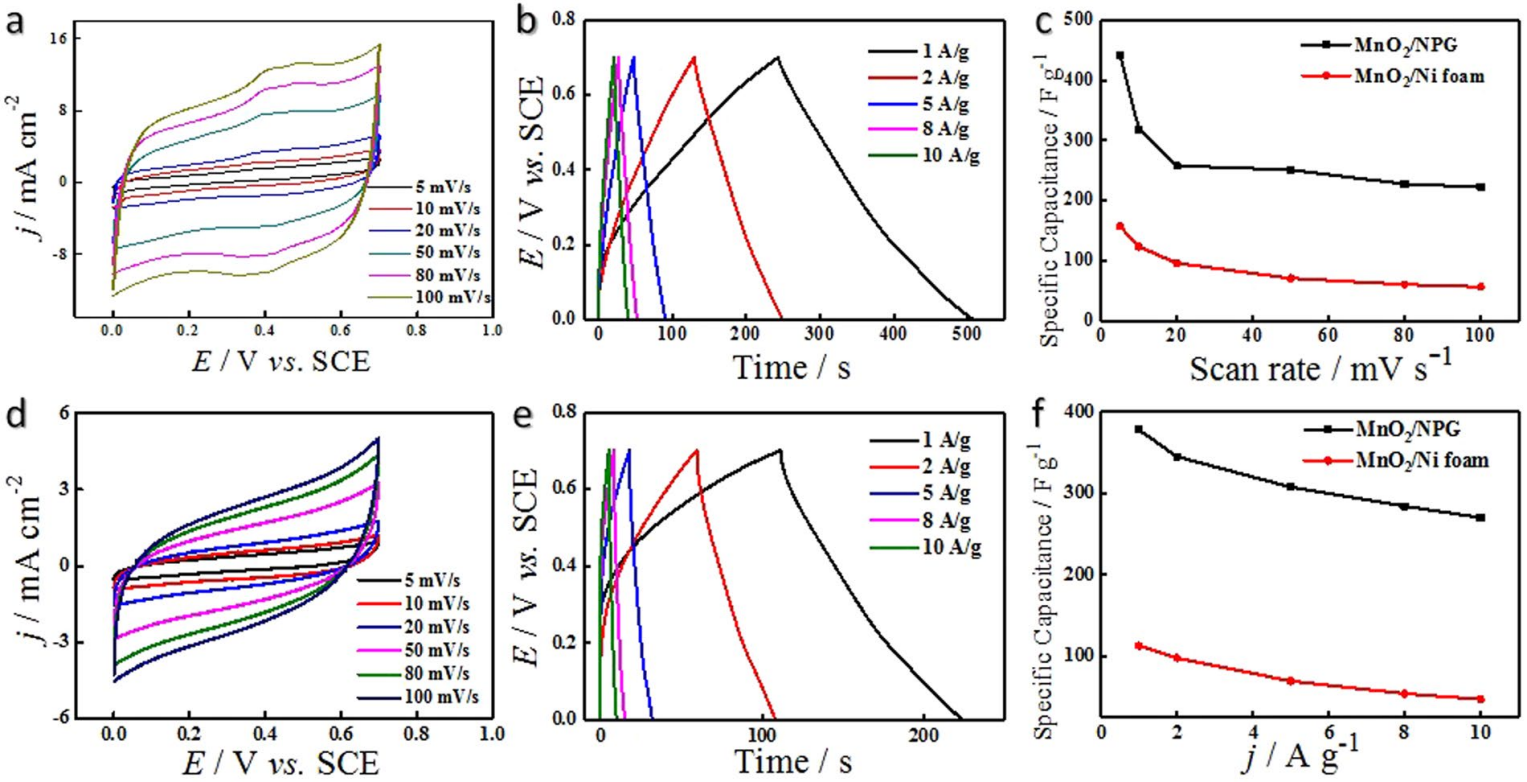
CV curves of the MnO_2_/NPG (**a**) and the MnO_2_/Ni foam (**d**) at different scan rates (5–100 mV s^−1^), GCD curves of the MnO_2_/NPG (**b**) and the MnO_2_/Ni foam (**e**) at different current densities (1–10 A g^−1^), and corresponding specific capacitance comparisons as a function of the scan rate (**c**) and the current density (**f**). All the tests above were performed in 1 mol/L Na_2_SO_4_ aqueous solution.

The electrochemical measurements of the MnO_2_/NPG and MnO_2_/Ni foam electrodes were tested in 1 mol/L Na_2_SO_4_. Typical CV curves of the MnO_2_/NPG electrode at scan rates ranging from 5 to 100 mV s^−1^ show much better rectangularity than that of the MnO_2_/Ni foam electrode (Fig. 3a,d). It is obviously seen that the current intensity increases with the scan rates, while the positions of the redox peaks shift slightly^6^, showing its good electrochemical reversibility. Generally, the energy storage process of MnO_2_ is a reversible successive surface redox reaction, resulting the shape of CV curves similar to the electric double layer capacitor (EDLC)^37^, which also can be showed in Fig. 3d. However, the radius of electrolyte ions and the microstructure of the MnO_2_ can possibly lead to faradic phenomena occur during the charge-storage mechanism, which is not so successive. As described in several previous researches, the microstructure of the MnO_2_@NPG/Ni foam has a big difference with MnO_2_/Ni foam, which can make the intercalation/deintercalation of ions in the MnO_2_ solid phase possible and result in the redox peaks in CV curves^32,38–40^.

The higher current densities obtained by the MnO_2_/NPG show improved electron transportation and lower internal resistance. The superior performances of the MnO_2_ loaded on the NPG/Ni foam can be attributed to two main reasons: (1) the large contact area between the current collector and active material can significantly shorten the electron transfer distance and increase the number of electrochemically active sites for the redox reaction and (2) dispersing capacitive materials onto a large surface area greatly improved the conductivity of pseudocapacitive materials^12^.

Galvanostatic charge-discharge (GCD) curves were performed in Fig. 3b,e. The GCD curves of the MnO_2_/NPG, with current density ranging from 1 A g^−1^ to 10 A g^−1^, are more symmetrical than those of the MnO_2_/Ni foam, which validate its improved capacitive behavior. In addition, at the start of the discharge curves, the voltage drop is quite small, indicating very low internal resistance between the electrodes.

For specific capacitances at various scan rates, the MnO_2_/NPG electrode showed an increase of approximately 3 times when compared to the MnO_2_/Ni foam electrode, as is shown in Fig. 3c. The *C*_*v*_ decreases with an increase of the scan rates, and the highest specific capacitance of the MnO_2_/NPG electrode reached upwards of 442 F g^−1^ at the scan rate of 5 mV s^−1^. The consistency of these curves reveals the excellent capacitive behavior of the MnO_2_/NPG electrode. Besides, this capacitance of this MnO_2_/NPG electrode was compared with some previous researches based on MnO2 material, demonstrating rather good performance, as presented in Table 1.

**Table 1:** Capacitance comparison between this work and other previous MnO_2_ based materials.

Electrode	Specific capacitance/F g^−1^	Current density/A g^−1^	Scan rate/mV s^−1^	Reference
MnO_2_/Ni foam	325	—	5	^41^
MnO_2_/nanoporous silver	384	1	—	^42^
MnO_2_/Au core-shell	524	0.56	—	^43^
Porous MnO_2_ tubes	365	0.25	—	^15^
Al doped MnO_2_	213	0.1	—	^44^
MnO_2_@Graphene	130	—	2	^45^
SWNTs@MnO_2_/polypyrrole	351	—	1	^17^
**MnO**_**2**_ **/NPG**	378	1		This work
**MnO**_**2**_ **/NPG**	442		5	This work

**Figure 4:**
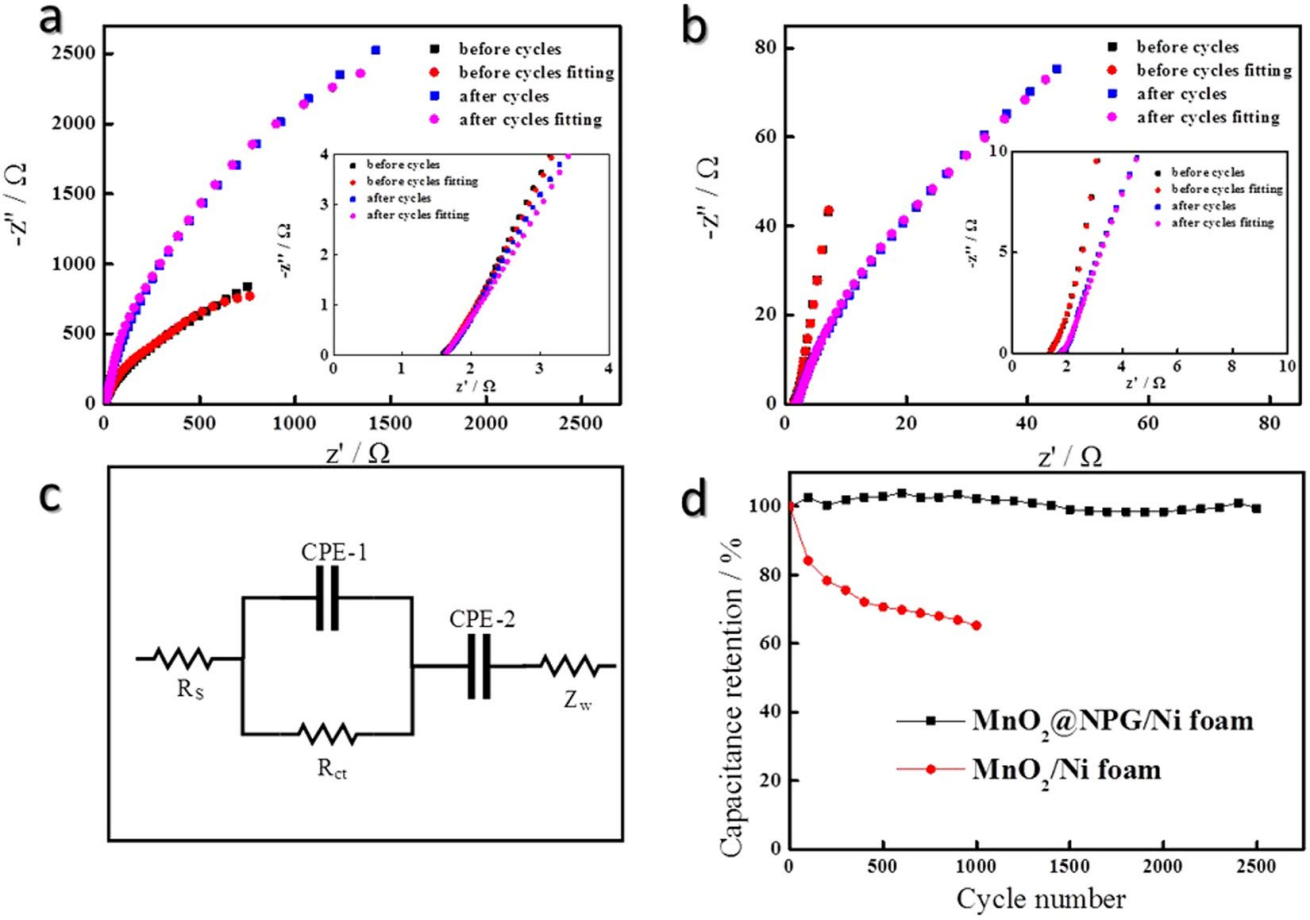
Nyquist plots of EIS performed in 1 mol/L Na_2_SO_4_ for the MnO_2_/NPG before and after 2500 cycles (**a**) and the MnO_2_/Ni foam before and after 1000 cycles (**b**), the equivalent circuit diagram of the two electrodes for the EIS analysis (**c**), and cycling performance of the corresponding electrodes during 2500 cycles and 1000 cycles at the scan rate of 50 mV/s (**d**).

The enhanced electrochemical performance of the MnO_2_/NPG hybrid electrode was further confirmed by the electrochemical impedance spectroscopy (EIS) measurements. Figure 4a and b shows the Nyquist plots for the MnO_2_/NPG and the MnO_2_/Ni foam after the 2500th and 1000th cycles respectively. The equivalent electrical circuit in Fig. 4c was obtained by fitting the impedance data. The internal resistance (*R*_*s*_) is the sum of the ionic resistance of the electrolyte, the intrinsic resistance of active materials, and the contact resistance at the active material/current collector interface. The Faradic reactions corresponded to the interfacial charge transfer resistance (*R*_*ct*_), which are related to the interface between the electrode and electrolyte, and the electrical charge transfer in the Faradic process of the electrode materials. A constant phase element was used to account for the double-layer capacitance and pseudocapacitance. The Warburg impedance corresponds to the straight line in low-frequency area, which is associated with the ion diffusion in the electrode. The charge-transfer resistances obviously decreased with nanoporous gold coating, which reveals that the bi-continuous nanoporous network of the MnO_2_/NPG electrode significantly improved the conductivities of the electrode materials, ion transfer, and charge transfer.

After cycles, the *R*_*ct*_ of the MnO_2_/NPG electrode decreased from 95.56 Ω to 23.59 Ω where the MnO_2_/Ni foam electrode evidently increased from 543.1 Ω to 3541 Ω. The increased resistance of the MnO_2_/Ni foam is mainly attributed to the low conductivity of MnO_2_, compared with the as prepared MnO_2_. MnO_2_ sheets after cycles become larger and thicker after 1000 cycles, which causes lower electron/ion transfer and thereby capacitance fading of pure MnO_2_ due to the reduced effective surface areas and low electronic conductivity. To prove these, SEM images of MnO_2_/NPG electrode and MnO_2_/Ni foam electrode after cycling 2500 time and 1000 time were shown in Figure S1. As seen in the SEM images, after 1000 cycles, most of the MnO_2_ sheets loaded directly on Ni foam agglomerate tightly, which could be the main reason for the increase of R_ct_ (Figure S1a–c). However, the structure of the MnO_2_@NPG/Ni foam electrode retained well and distribution of MnO_2_ sheets seems more uniform, which contributes to the decrease of the R_ct_ (Figure S1d–f). Compared to the bare MnO_2_ micro-supercapacitor, the MnO_2_/NPG composite micro-supercapacitor has a lower resistance, which is of great importance since less energy and less power will be wasted to produce unwanted heat during the charge–discharge processes.

Furthermore, the cycling performances of the MnO_2_/NPG electrode and the MnO_2_/Ni foam electrode after long-term cycling are shown in Fig. 4d. The specific capacitance of the MnO_2_/Ni foam electrode rapidly dropped to 65.3% after 1000 cycles, as a result of irreversible reactions. Instead, the MnO_2_/NPG electrode was found to exhibit an excellent cycle life over the entire cycle-number range. The capacitance retention of the MnO_2_/NPG electrode is quite stable and still remains 99% of its initial value even after 2500 cycles, indicating that the electrode materials had excellent cycle stability and quite a high degree of reversibility in charge-discharge cycling.

## Discussion

In this work, we have prepared the MnO_2_/NPG hybrid electrode by electrodeposition of a gold-tin alloy on Ni foam, selective chemical etching in alkaline media, and electrodeposition of MnO_2_. This 3D nanoporous substrate with high porosity greatly enhances the surface area compared to that of the planar electrode and improves the conductivity and dispersibility of the loaded MnO_2_. Moreover, the MnO_2_/NPG hybrid electrode shows remarkable enhancements in specific capacitance, charge-discharge ability, as well as cyclic stability. The simplicity of the nano-architectured electrodes and their excellent performances has shown promising features for practical energy storage systems. The as prepared MnO_2_/NPG hybrid electrodes exhibit a specific capacitance of 442 F g^−1^ at a scan rate of 5 mV s^−1^. The specific capacitance only decreased by 1% after 2500 cycles at a scan rate of 50 mV s^−1^. Meanwhile, a high power density of 3513 W kg^−1^ and an energy density of 25.73 Wh kg^−1^ were achieved. By comparing the properties with those of MnO_2_/Ni foam electrodes, the NPG structure has shown its importance and advantages in our hybrid electrodes.

## Methods

### Chemicals and materials

The Au-Sn plating solution was purchased from Huizhou Leadao Electronic Material Co. Ltd. Hydrochloric acid (HCl), potassium hydroxide (KOH), hydrogen peroxide (H_2_O_2_), sodium sulphate (Na_2_SO_4_) and dimethyl sulfoxide (DMSO) was purchased from Guangzhou Chemical Reagent Factory. Ammonium acetate (NH_4_Ac) and manganese acetate (MnAc_2_) was purchased from Shanghai Aladdin Biochemical Technology Co., Ltd. Nickel foam was purchased from Kunshan Longshengbao Electronic Material Co., Ltd. Platinum foil electrode and saturated calomel electrode (SCE) were purchased from Shanghai INESA Scientific Instrument Co., Ltd.

### Fabrication of MnO_2_/NPG electrodes

**Figure 5:**
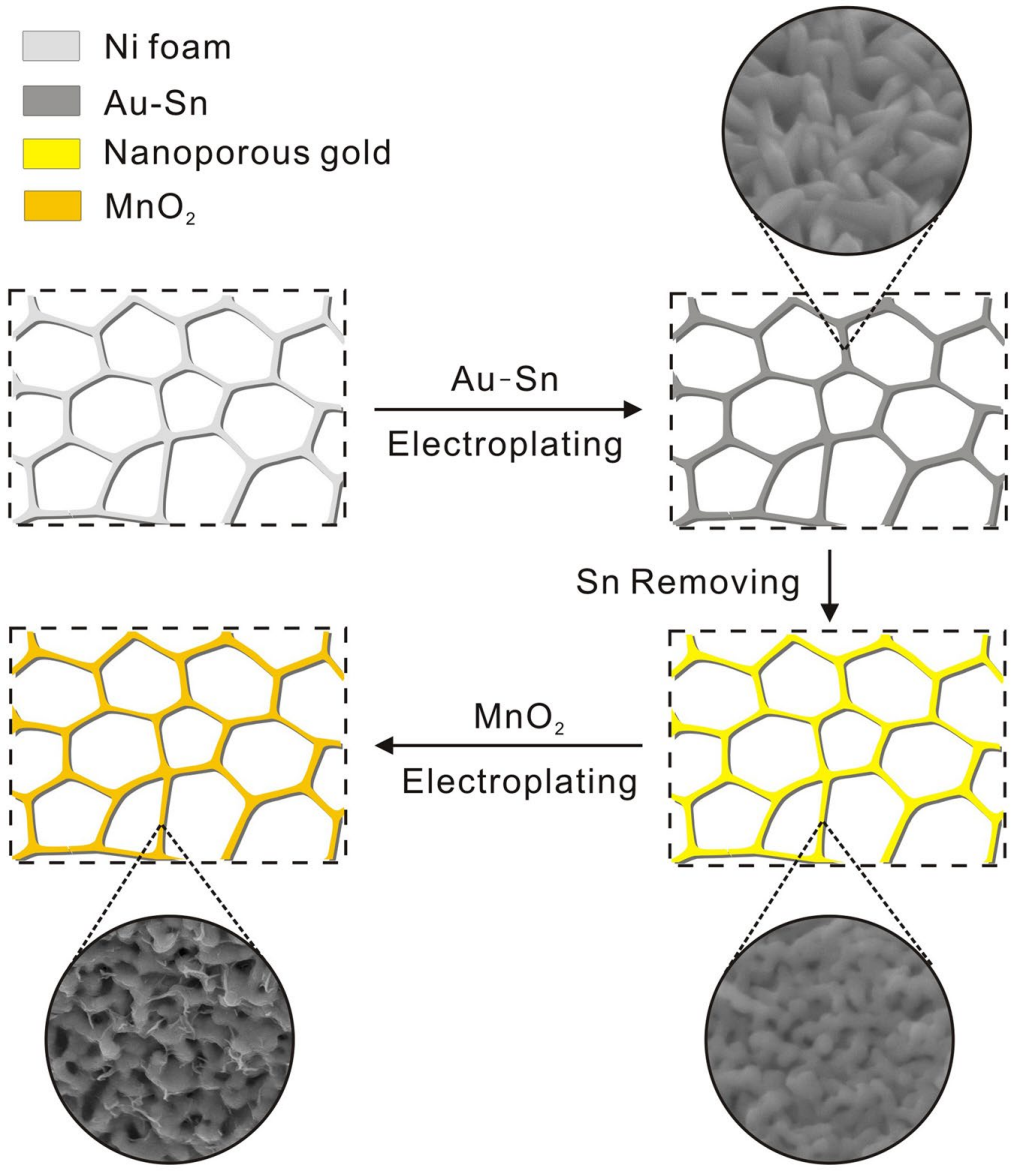
Schematic showing the fabricating procedure of the MnO_2_/NPG electrode.

The fabrication procedure consists of four main steps. As illustrated in Fig. 5, Ni foam was cut into the proper size (approximately 1 cm × 7 cm × 1 mm), pre-treated with 5 mol/L HCl solution for 30 min to remove the oxide layer on the surface, and then rinsed thoroughly with deionized water. The Au-Sn alloy electrodeposition was carried out in a two-electrode system with the clean Ni foam as the working electrode and a Pt foil as the counter electrode. The Au-Sn alloy film was galvanostatically electrodeposited on Ni foam in an Au-Sn alloy plating solution (Huizhou Leadao Electronic Material Co. Ltd., China) with a current density of 0.5 A dm^−2^ for 10 min at 45 °C.

After electrodeposition, the electrode was rinsed with deionized water and dried in air. It was then immersed into a 5 mol/L NaOH and 1 mol/L H_2_O_2_ solution for 3 days to selectively etch Sn away from the Au-Sn alloy film, leading to the formation of an NPG film on Ni foam. After etching, the Ni foam was carefully rinsed with deionized water and dried in air. Finally, MnO_2_ was electrodeposited on the NPG/Ni foam electrode from an aqueous solution containing 0.01 M manganese acetate (MnAc_2_) and 0.02 M ammonium acetate (NH_4_Ac) in a solvent mixture of 90% DI water and 10% dimethyl sulfoxide (DMSO) by a galvanostatic electrodeposition method. The MnO_2_ electrodeposition experiments were performed in a standard three-electrode electrochemical cell. The NPG/Ni foam electrode was used as the working electrode, a Pt foil as the counter electrode, and a saturated calomel electrode (SCE) as the reference electrode. The MnO_2_ electrodeposition was conducted by applying a constant current of 0.8 mA cm^−2^ for 20 min. As a control experiment, a MnO_2_/Ni foam electrode, without NPG film, was produced by the same procedure.

### Characterization methods

The microstructure of the samples was investigated using field-emission scanning electron microscopy (SEM, JEOL, JSM-6700F, 15 keV). X-ray diffraction (XRD) measurements were performed on a Rigaku D/max-2200/PC diffractometer using Cu Kα radiation. The amount of loaded MnO_2_ was detected by inductively coupled plasma mass spectrometry (ICP-MS, Thermo Scientific iCAP Qc). Electrochemical measurements were carried out on a Gamry Reference 600 electrochemical workstation in a three-electrode setup with 1 mol/L Na_2_SO_4_ as the electrolyte. The MnO_2_/NPG electrode, platinum foil, and a SCE electrode acted as the working electrode, counter electrode, and reference electrode respectively. The electrochemical impedance spectroscopy (EIS) measurements were conducted in a frequency range from 100 kHz to 0.1 Hz with a perturbation amplitude of 5 mV. All electrochemical measurements were performed at room temperature (25 ± 2 °C).

### Calculation methods

Voltammetric specific capacitances (*C*_*v*_, F g^−1^) were calculated from the CV curves at different scan rates by the following equation:

1 $${C}_{v}=(\int I\times dV)/(m\times s\times V)$$

where *I* is the current (A), *V* is the potential (V), *m* is the mass (g) of the loaded MnO_2_, and *s* is the scan rate (V s^−1^).

Charge-discharge specific capacitances (shown in Fig. 3f) were also calculated from the GCD curves with different current densities using the following equation:

2 $$C=(I\times {\rm{\Delta }}t)/(m\times {\rm{\Delta }}V)$$

where *I/m* is the current density (A g^−1^) applied in charge/discharge measurements, *∆t* is the discharge time (s), and *∆V* is the potential change (V).

## Electronic supplementary material

Supplementary Information
