# Supplementary material for: Three-Dimensional Bi-Continuous Nanoporous Gold/Nickel Foam Supported MnO2 for High Performance Supercapacitors
Source: Sci Rep. 2017 Dec 19;7:17857. doi: 10.1038/s41598-017-17872-3 (PMC5736659; doi:10.1038/s41598-017-17872-3)
Supplement: Supplementary file 1 — Supplementary Information [file 41598_2017_17872_MOESM1_ESM.doc]

Supporting Information for

Three-Dimensional Bi-Continuous Nanoporous Gold/Nickel Foam Supported MnO_2_ for High Performance Supercapacitors

**Jie Zhao ^1^, *, Xilai Zou ^2, 3^, Peng Sun ^2, 3^ , Guofeng Cui ^2, 3^, ***

^1^School of Mechanical and Automotive Engineering, South China University of Technology, Guangzhou, 510640, China. ^2^Key Laboratory for Polymeric Composite & Functional Materials of Ministry of Education, School of Chemistry, Sun Yat-sen University, Guangzhou, 510275, China. ^3^Key Laboratory of Low-carbon Chemistry & Energy Conservation of Guangdong Province, Sun Yat-sen University, Guangzhou, 510275, China. Correspondence and requests for materials should be addressed to J. Z. (email: zhaoj77@scut.edu.cn) or G. F. C. (email: cuigf@mail.sysu.edu.cn)

**
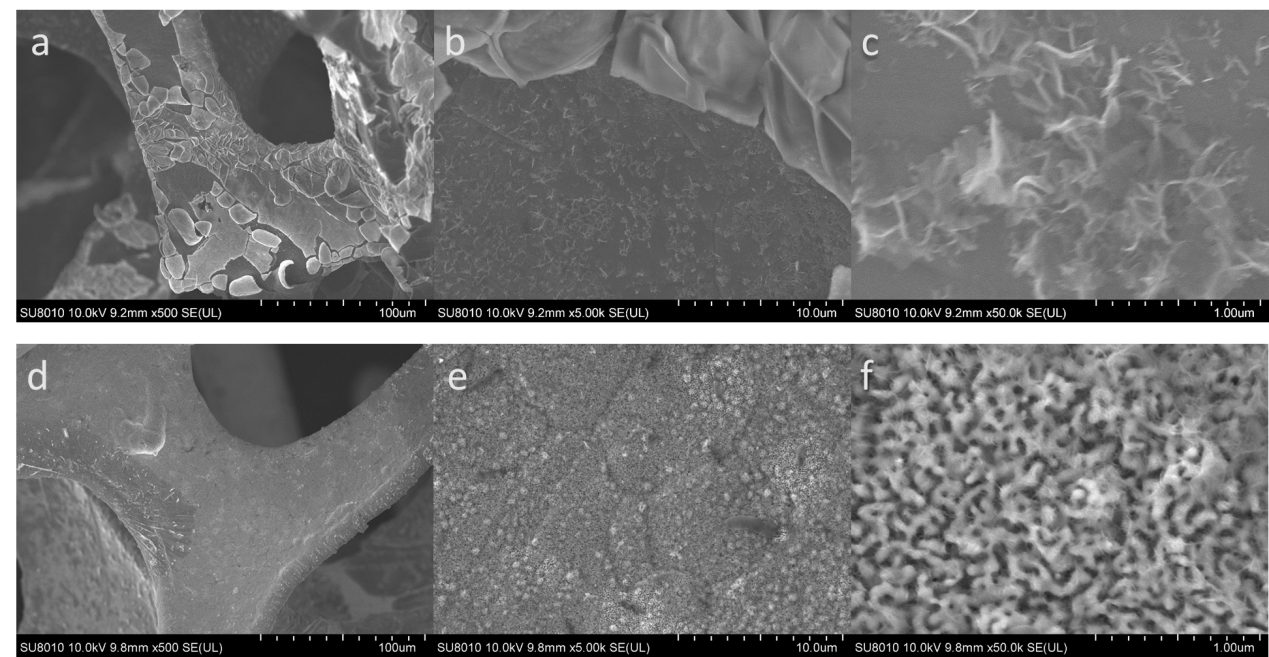
**

**Figure S1** SEM images of the MnO_2_/Ni foam (a) to (c) and the MnO_2_/NPG/Ni foam (d) to (f) after cycling corresponding times.

**
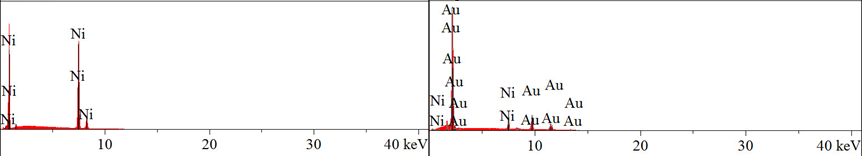
**

**Figure S2** EDS spectrum of Ni foam (left) and NPG/Ni foam (right).
